# Technical Difficulties Associated with the Formation of Carbon-11 Labelled Carboxylic Acids

**DOI:** 10.6028/jres.093.068

**Published:** 1988-06-01

**Authors:** Patricia Landais, Ronald Finn

**Affiliations:** National Institutes of Health, Clinical Center, Department of Nuclear Medicine, Bethesda, MD 20892

Positron Emission Tomography (PET) is an established method for probing biochemical processes in vivo and holds promise for being an established clinical tool [1–4], PET produces crosssectional tomographic images which monitor fundamental physiologic, biochemical and pharmacological processes such as glucose metabolism, oxygen utilization, blood flow, amino acid and fatty acid transport and metabolism, tissue receptor and drug binding characteristics. PET is similar to x-ray Computed Tomography (CT) and Magnetic Resonance Imaging (MRI) in that it is a computed tomographic technique. Unlike these clinical techniques, PET requires the injection of a positron emitting compound of particular physiologic interest. At present, the positron emitting radionuclides commonly being investigated include carbon-11 (*t*_1/2_=20.4 m), nitrogen-13 (*t*_1/2_ = 9.98 m), oxygen-15 (*t*_1/2_ = 123 s) and fluorine-18 (*t*_1/2_= 109.8 m). The restrictions imposed upon the pharmaceutical chemist due to the physical half lives of these radionuclides mandate not only a sensitive, but also a rapid quality assurance program for the finished formulations. The preparation of a class of fatty acids is an excellent illustrative example of the technical difficulties and chemical subtleties associated with the research effort on short-lived radiopharmaceutical diagnostic compounds.

Biologically, long chain fatty acids are important substrates utilized to meet the myocardial energy requirements. It is theorized that PET might provide insight into changes in the utilization of these compounds as a result of therapies or organ dysfunction caused by disease. Moreover, an added benefit of the successful synthesis of particular fatty acids is that it allows the radiopharmaceutical chemist the option of preparing other synthons, such as acid chlorides, aldehydes and alcohols.

Fatty acids labelled with radio-carbon are prepared from the corresponding organomagnesium halide. Although numerous fatty acids have been prepared utilizing this synthetic approach, few detailed reports exist. For this reason we now report our results on the preparation of (^11^C)-cyclopropanecarboxylic acid and (^11^C)-3,4-dimethoxybenzoic acid. These compounds are required as starting reagents for the preparation of (^11^C)-cyclofoxy [1,2] and (^11^C)-dopa [3,4] respectively.

## Experimental

“No carrier added” (^11^C)-carbon dioxide is produced in high radionuclidic and radiochemical purity by the ^14^N(p, α)^11^C nuclear reaction on a nitrogen gas target using the Japan Steel Works Limited Baby Cyclotron (BC-1710). The ^11^CO_2_ is condensed from the nitrogen gas target stream on a vacuum line using a glass radiator trap cooled to −193 °C. Following complete removal of the nitrogen gas, the ^11^CO_2_ is allowed to react with freshly prepared organomagnesium halide. After 2–3 minutes, the reaction is quenched by acid hydrolysis. In the experiments involving the addition of carrier, known quantities of carbon dioxide were added to the cyclotron produced product.

A catalytic concentration of 1,2 dibromoethane was used to initiate the reactions [6], The concentration of the alkyl magnesium halide formed, with suitable correction made for non-Grignard basicity [5], was determined by acidimetrie titration using CCI_4_. Moreover, radio thin layer chromatography was used to establish the radiochemical purity of the synthesized fatty acid.

**Figure 1:**
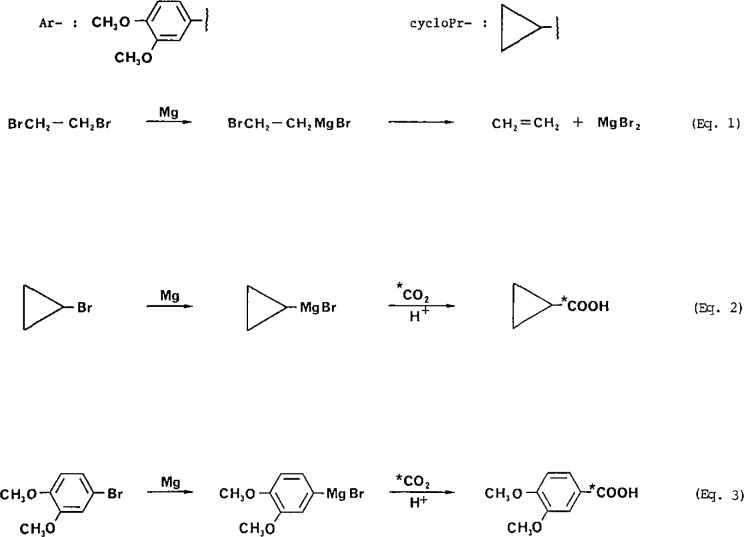
Activation of magnesium by entrainment eq (1) and reactions leading to the formation of radiolabelled cyclopropanecarboxylic acid eq (2) and 3,4-dimethoxybenzoic acid eq (3).

The chemical scheme and specific reactions leading to the products are shown in figure 1 and our analytical results are summarized in table 1.

## Discussion

The reaction of Grignard reagents with radiolabelled carbon dioxide has proven to be an effective means of preparing labelled carbonyl compounds. The state of the magnesium surface has a significant impact upon the yield of the reaction and subsequent “side” reactions. It is important to use high chemical purity magnesium with a large surface area for the preparation of the organomagnesium reagent. The principal competing reactions that lead to a reduced yield of Grignard reagent are a) a coupling (Wurtz) reaction and b) a disproportionation reaction. These reactions are favored by elevated temperatures and excess halide concentration. Our experience indicates that the formation of the Grignard reagent proceeded smoothly when the alkyl bromide concentration was greater than 0.15 mol/L. The freshly prepared Grignard reagent exhibits a high chemical reactivity. Efforts to exclude oxygen, moisture and stable carbon dioxide are important to maintain the high specific activity and to minimize “side” reactions. Only freshly distilled solvents, flame-dried magnesium turnings and glassware, and an inert atmosphere are appropriate.

The radio thin layer chromatography shows that several radioactive products are formed which lead to lower chemical yield of the labelled fatty acid. These products could be the result of competing reactions resulting in ketone formation or alcohol formation from impure or excess Grignard reagent. In the preparation of “no carrier added” radiopharmaceuticals side product formation is generally limited by restricting reaction times.

In conclusion, the preparation of “no carrier added” carbon-11 labelled fatty acids is readily achievable utilizing organomagnesium compounds and carbon dioxide labelled with carbon-11; however, impure reagents and experimental conditions may reduce the concentration of the desired Grignard reagent. To find application in PET, the preparation of both radionuclidic and radiochemically pure compounds is of paramount concern. In the case of radiolabelled carboxylic acids, care must be exercised in determining the yield of Grignard reagent formation based solely upon assay of nonisolated intermediates.

## Figures and Tables

**Table 1:** Formation yields of magnesium compounds and the corresponding carboxylic acids.

	Grignard reagent			Carboxylic acid	
	R′X	Ar-MgBr/Ar-Br	cycloPr-MgBr/cycloPr-Br	Ar-COOH	cycloPr-COOH

Experimental Yield	–	59 (32–100)*	71 (52–100)		
		
	BrCH_2_–CH_2_Br	40 (7–76)	41 (8–71)	42 (25–60)	52 (10–66)
		
	CCI_4_	–	55 (42–66)		

Published Yield		–	80	95<	60–80

* The numbers in parentheses represent the extremes in our experimental results.
